# 400 W average power Q-switched Yb:YAG thin-disk-laser

**DOI:** 10.1038/s41598-022-20917-x

**Published:** 2022-10-08

**Authors:** Saeid Radmard, Ahmad Moshaii, Kaveh Pasandideh

**Affiliations:** 1 Department of Physics, Tarbiat Modares University, P.O Box 14115-175, Tehran, Iran; 2 Iranian National Center for Laser Science and Technology, P.O Box 14665-576 Tehran, Iran

**Keywords:** Applied physics, Atomic and molecular physics, Optical physics

## Abstract

We report on producing up to 403 W average power directly from an acousto-optically Q-switched Yb:YAG thin-disk laser (TDL). To achieve this power, it has theoretically and experimentally been shown that the laser stability border could be shifted toward higher repetition rates by engineering of the output coupler transmittance. This allows for stable operation of the laser at higher frequencies and a further increase in the power extraction from the active medium. Using an output coupler with 93% reflectivity, a maximum average power of 403 W at the repetition rate of 12.0 kHz has been recorded under the pump power of 1220 W. Furthermore, the maximum pulse energy of 57 mJ was produced at the repetition rate of 1.00 kHz and the pump power of 520 W. The characteristics of the laser at various Q-switching rates and the pump powers have been investigated. In addition, a numerical study for supporting the experimental results has been proposed here. To the best of our knowledge, the achieved average power and the pulse energy are the highest values reported to date from a Q-switched Yb:YAG TDL. The results pave the way to further power scaling of solid-state Q-switched oscillators.

## Introduction

Thin-disk-lasers (TDLs) are a class of relatively low-cost and high-average power laser sources^1^. The unique specifications of these lasers in power and beam quality made them very attractive for producing both CW and pulsed laser systems^2^. Achieving a promising optical efficiency of 80% makes them more favorable for industrial applications^3^. The high average power TDLs with µs to ns pulse duration^4,5^, ultrafast pulses^6,7^ and high average power green TDLs^8^ have been reported. TDL devices with average powers of more than 10 kW in CW mode and several hundred watts in pulsed operation have been commercialized^9^.

In the high power operation, Q-switching, cavity dumping and oscillator-amplifier setup are the three main pulse generation methods in the µs or ns region^10^. Despite initial efforts to utilize these methods^11–13^, in the TDLs, cavity-dumping has been commonly used for pulse generation in this region, with average powers up to several hundreds of Watts^5,14–16^. However, it has some important drawbacks including high voltage driving, relatively high cost of the dumping elements, and the broad-spectrum^17^. On the other hand, Q-switching is a common way for generating pulsed solid-state lasers by using Acousto-Optical (AO) or Electro-Optical (EO) modulators. Compared to EO Q-switching and cavity-dumping, the AO Q-switching is attractive because it doesn’t need any high voltage and polarizing elements in the resonator, so it is less complicated and more economical^10^.

The average power of pulsed lasers is very important in industrial applications since it directly determines the processing speed^18,19^. In rod oscillators, the maximum average power is restricted by the fracture limit of the active medium^20,21^. Meanwhile, the thermal effects destroy the laser beam quality, so achieving higher average powers requires various amplification stages^22,23^. Alternatively, nonlinear effects and fiber damage are the main factors that challenge the power scaling of pulsed fiber lasers^24,25^. However, due to the active medium geometry, TDLs are less influenced by the above-mentioned restricting factors, and scaling-up of the average power at constant pump power density is realized^1^.

Noticeably, in Q-switched TDLs, two main factors restrict the scaling up of the output average power^26^. Both of these factors originate from the low gain coefficient of the active medium. The output coupler (OC) reflectivity typically is near one, so the cavity-internal-energy is high enough to damage the disk even for output pulses in the order of hundred mJ. Therefore, the laser repetition rate should be increased to enhance the average power. However, this could lead to strong output pulse energy fluctuation and the appearance of pulse instabilities^13,27^. This instability originates from the dynamic of Q-switched lasers and is still the subject of experimental and theoretical studies, also in other kinds of lasers^26,28,29^. Although pulse instability is an intrinsic property of the Q-switched lasers, it is expected to appear more pronouncedly in TDLs because of their low gain factor^28^. Active feedback controlling technologies could be implemented to stabilize the laser output in this region but add further complexities to the laser and limit their flexibility^16,30^.

Among the related papers on high power Q-switched TDLs, there is no comprehensive study on scaling up the average power of Q-switched Yb:YAG TDLs, or characterization of the pulse’s instabilities dependency on the laser design parameters. In almost all of these reports, the generated pulses have been reported under normal operational conditions, typically at low average powers^11,13^.

The main goal of this study is to produce about 400 W average power from a Q-switched Yb:YAG TDL, using a commercial AO modulator and a simple V-shaped resonator. We have shown that the laser can stably operate at higher repetition rates by engineering the output coupler transmittance. This leads to the enhancement of the achievable average power from the disk. In this way, by taking care of the damage threshold of the disk media by controlling the laser pulse fluency and the instability region of the laser, we have optimized this laser system to achieve a new record of the average power. Moreover, a numerical simulation has been presented to support the experimental results.

### Experimental setup

**Figure 1:**
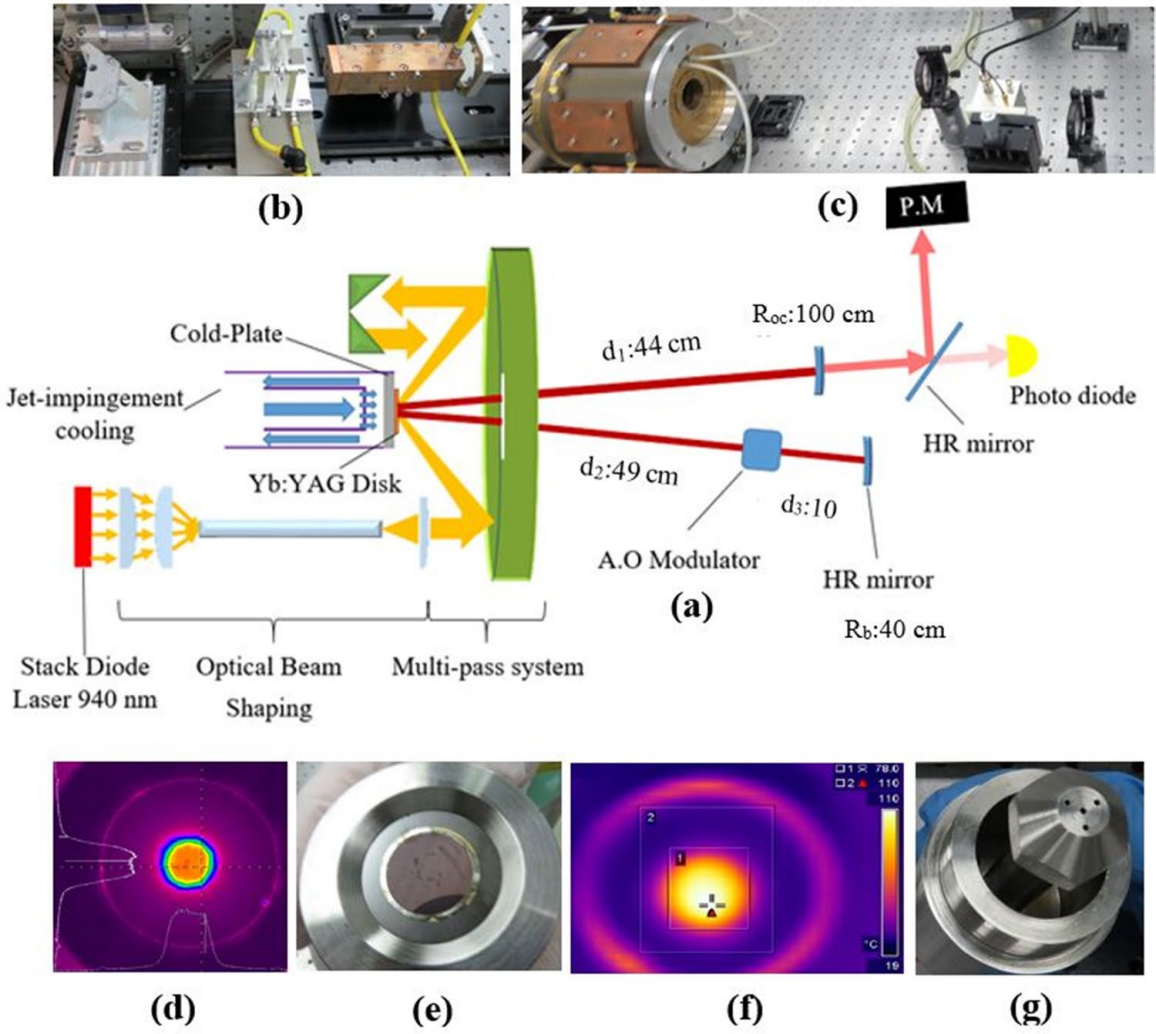
Overview of the experimental setup: (**a**) schematic of the designed laser, (**b**) beam-shaping system elements, (**c**) multi-pass pump module and the resonator, (**d**) pump profile on the disk, (**e**) the bonded disk on the Cu–W cold-plate, (**f**) the temperature distribution on the disk surface and (**g**) the jet-impingement cooling system.

In Fig. 1, a depiction of the designed laser setup can be seen. The pumping is provided by a high-power stack diode laser at 940 nm with output power up to 1300 W. Due to the importance of the pump profile in laser performance, an optical beam-shaping system has been designed to produce a uniform and near top-hat shape pump profile with desired spot diameter on the disk surface^31^. The system uses a light pipe to homogenize the stack’s output profile, cylindrical lenses to compensate for the diode laser’s optical astigmatism, and some collimating lenses to adjust the pump beam size on the disk (all shown in Fig. 1b).

A multi-pass pumping system is used to increase the path length of the pump beam in the disk media and increment of the pumping efficiency^32^. The designed multi-pass system, which is shown in Fig. 1c, contains a parabolic mirror with a focal length of 100 cm and folding-mirrors that redirect the unabsorbed pump beam onto the disk media. This multi-pass pumping system provides 20 single-pass crossing through the disk, with about 90% absorption efficiency. Eventually, as shown in Fig. 1d, a top-hat pump profile with a spot diameter of 6.5 mm is formed on the disk.

The disk media was a commercial Yb:YAG crystal with a doping concentration of 9% and a thickness of 180 µm. The disk is a high-transmission coated for the wavelengths of 940 nm (the pumping wavelength) and 1030 nm (the lasing wavelength) at the front side and high reflection coated for both wavelengths at the backside. The backside of the disk was bonded on a Cu–W plate with an Au–Sn solder to provide an efficient heat-transfer media for water-cooling of the disk. A picture of the bonded disk and the uniform temperature profile on the disk surface under the pumping is shown in Fig. 1e,f, respectively. For efficient heat dissipation from the Yb:YAG disk, under the influence of the pumping spot, a jet impingement water cooling system^33^, shown in Fig. 1g, was designed and implemented.

Also, as shown in Fig. 1c, a simple V-shape resonator has been designed so that the stability factor, *g*_1_*g*_2_, remains between 0.47 and 0.54, depending on the pumping power. Notably, due to the thermal bending, the disk has a concave curvature with a radius between 2.2 and 4.1 m. This variable dioptric of the disk was considered in the resonator design.

For Q-switching, an acousto-optical modulator was put in the back arm of the resonator. The modulator contains a BBO crystal with a length of 5 cm and a clear aperture of 5 mm, a piezoelectric transducer, and an RF driver. A fast Si photodetector and a digital oscilloscope recorded the pulse train during the experiments.

### Theoretical analysis

For modeling the laser operation and the dynamics of the output pulses, a numerical analysis based on the laser rate equations and the loss-gain equilibrium condition was applied. In Yb:YAG, the lifetime of sublevels is too short; hence it is reasonable to consider a two-energy-level system for modeling the laser mechanism in this active medium^34^. Considering the absorption/emission at the pumping/lasing wavelength, the following coupled differential equations can be applied^35^

1 $$\begin{aligned} \frac{{dN_{2} }}{dt} = & - \frac{{N_{2} }}{{\tau_{f} }} + \frac{{I_{p} \,\eta_{abs} }}{{h\,v_{P} \,l_{d} \,}} - \frac{{M\,I_{r} }}{{h\,v_{L} }}\left( {N_{2} \,\sigma_{em}^{L} - \left( {N_{tot} - N_{2} } \right)\sigma_{abs}^{L} } \right) \\ \frac{{dI_{r} }}{dt} = & \left( {M\,l_{d} \,\left[ {N_{2} \,\sigma_{em}^{L} - \left( {N_{tot} - N_{2} } \right)\sigma_{abs}^{L} } \right]\,\,I_{r} + \left[ {\ln (1 - loss_{{\text{int}}} ) + \ln (1 - T_{oc} )} \right]I_{r} } \right)\frac{c}{2L} \\ & + \frac{{dI_{f,eff} }}{dt}. \\ \end{aligned}$$

Here,$\tau_{f}$ is the upper-laser-level lifetime, *I*_*p*_ is the pump power density on the disk, *h* is Planck’s constant and ${v}_{L/P}$ is the frequency of laser/pump photons. Also, $N_{2}$ is the upper-laser-level population density, $I_{r}$ is intra-cavity power density, $l_{d}$ and ${N}_{tot}$ are the disk thickness and the Yb ions density in the active medium, respectively. The quantity ${\sigma }_{abs/em}^{L}$ is the absorption/emission cross-section at the laser wavelength. Also, $M$ is the number of passes of the laser radiation through the active medium during each round-trip in the resonator, and *L* is the effective length of the resonator. *T*_*oc*_ is the output coupler transmission, and *loss*_*int*_ represents the internal loss of the laser resonator. The absorption efficiency of the pump radiation, ${\eta }_{abs}$, for a ${M}_{p}$-passes pumping system is given by^36^

2 $$\begin{gathered} \eta_{abs} \, = \,1 - e^{{ - M_{p} \sigma_{abs}^{p} N_{dot} f_{B} l_{{d}} }} \hfill \\ f_{B\,} \, = \,1 - \frac{{\sigma_{abs}^{p} \, + \,\sigma_{em}^{p} }}{{\sigma_{abs}^{p} }}\,\frac{{N_{2} }}{{N_{tot} }}. \hfill \\ \end{gathered}$$

Also, the $dI_{f,eff} /dt$ describes the starting energy for the pulse build-up. ${I}_{f,eff}$ is the effective intensity of fluorescence radiation by the disk, obtained by

3 $$\frac{{dI_{f,eff} }}{dt} = \alpha \frac{{N_{2} }}{{\tau_{f} }}\,\frac{c}{2\,L}hv_{f} \,l_{d} .$$

Here, *α* is the part of fluorescence photons that is used efficiently in pulse build-up and was estimated by considering $M^{2}$ factor of the designed resonator, and the portion of fluorescence photons that are contributed to the laser oscillation^27^. The values of parameters used in the numerical analysis have been shown in Table 1.

**Table 1:** The values of parameters that were used in the numerical analysis.

Quantity	Magnitude
$\tau_{f}$	950 μs
$\upsilon_{L}$	$2.91\, \times 10^{14} \,{\text{Hz}}(\lambda = 1030\,nm)$
$\upsilon_{P}$	$3.19\, \times 10^{14} \,{\text{Hz}}\;(\lambda = 940\,nm)$
$l_{d}$	180 μm
$N_{dot}$	$1.21 \times 10^{21} \,{\text{cm}}^{ - 3} \;(dot:9\% \,at)$
$\sigma_{abs}^{p}$	$7.21\, \times 10^{ - 21} \,{\text{cm}}^{2}$
$\sigma_{em}^{P}$	$1.60\, \times 10^{ - 21} \,{\text{cm}}^{2}$
$\sigma_{abs}^{L}$	$1.25\, \times 10^{ - 21} \,{\text{cm}}^{2}$
$\sigma_{em}^{L}$	$2.07\, \times 10^{ - 20} \,{\text{cm}}^{2}$
$loss_{{\text{int}}}$	$0.014$
$M$	$4$
$L$	$106.5\;cm$
$\alpha$	$1.7 \times 10^{ - 9}$

To provide a theoretical investigation of the experimental results, the coupled Eqs. (1) and (2) were numerically solved using fourth-order Runge–Kutta method. The values of the parameters used in the numerical analysis are given in Table 1. A trapezoidal pulse train modeled the modulated loss of the laser resonator with a rise time of 100 ns.

## Results and discussion

The power measurement was carried out with two different power meters during the experiments. The first was used for the powers below 300 W, with a resolution of 0.1 W, and the second for more than 300 W, with a resolution of 1 W. The repetition rate of the pulse train was measured using a fast photodiode connected to an oscilloscope, and this system can measure the repetition rate with a resolution of much better than 1 Hz.

At higher repetition rates, the bifurcation in the energy of the laser output pulses was observed. In these conditions, there is the possibility of the formation of unexpected high energy pulses, which could damage the disk. Therefore, we considered a self-defined 10% variation in the pulse’s amplitude of a pulse train as the border of instability. This means that when more than 10% variation in the pulse’s amplitude was observed on the oscilloscope, the laser was considered unstable, and the corresponding pulse data was not reported. In addition, all experiments were performed under the condition that the intra-cavity power fluency on the disk surface was less than the laser-induced damage threshold (LIDT) (~ 3 J/cm^2^) of the disk. This avoids the potential damage to the disk.

Three different OCs with reflectivities of 90%, 93% and 95% were utilized in the experiments. As we will show, by engineering the OC reflectivity (R_OC_), the stable operation of the laser at higher repetition rates becomes possible. Hence, the extractable average power from the active medium increases. For clarity, throughout this paper, we have used the abbreviation f_rep_ for the repetition rate.

**Figure 2:**
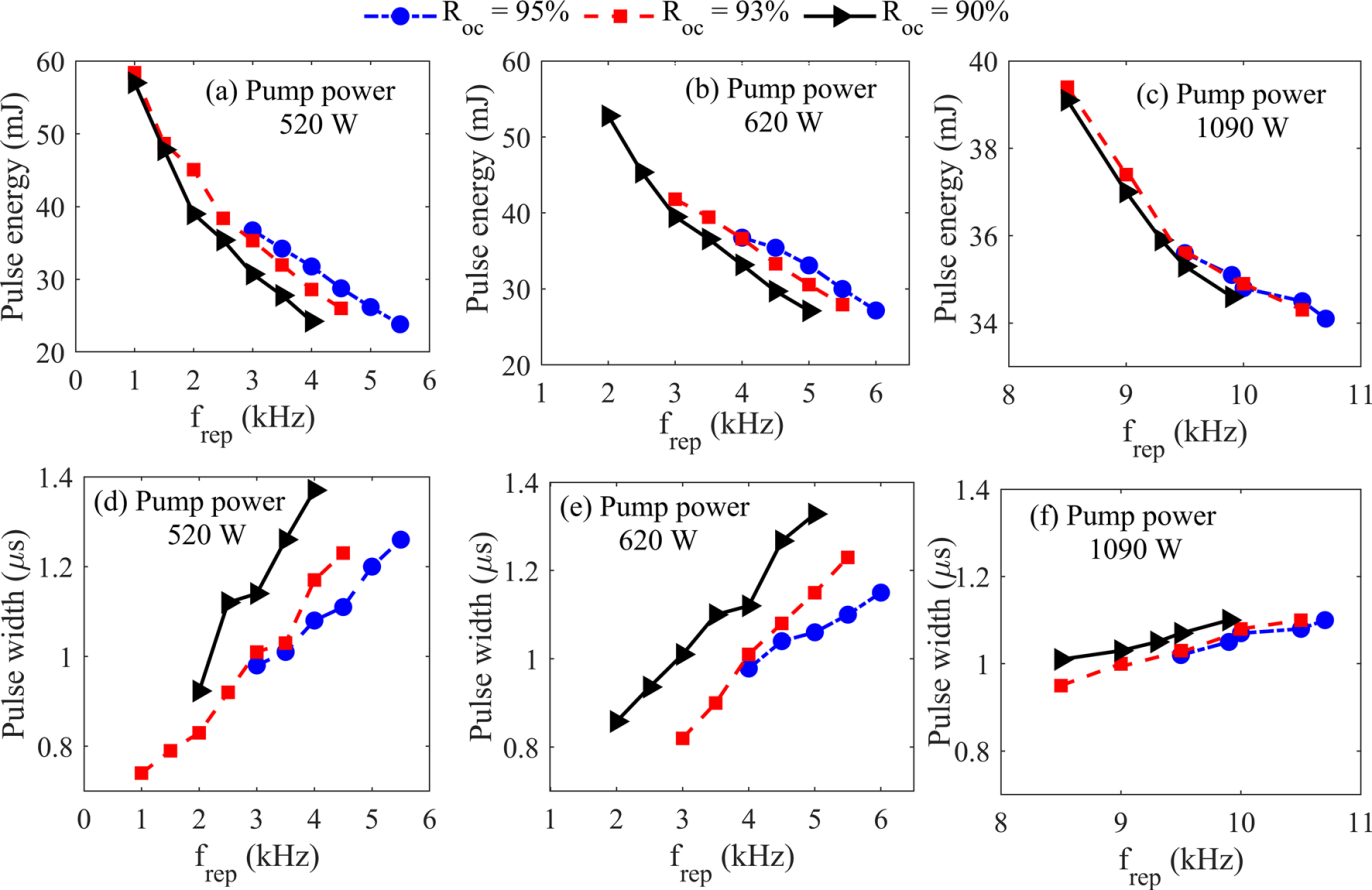
The energy and pulse width of output pulses as a function of repetition rate under the pump power of (**a,d**) 520 W, (**b,e**) 620 W, and (**c,f**) 1090 W.

Figure 2 shows the pulse energy and the time duration versus repetition rate for three different OC reflectivities. The experimental results for three different pump powers have been presented. For all pump powers, the energy of the output pulses decreases with the repetition rate. The highest pulse energy of 57.1 mJ was achieved at the repetition frequency of 1.00 kHz when the incident power was 520 W. It is considerably higher than previously reported pulse energy in Q-switched Yb:YAG TDLs^13^.

The results of Fig. 2 show that the dependency of pulse energy on the OC reflectivity is only pronounced at enough high repetition rates and for low pump powers. The pulse width increases with the repetition rate for all pump powers and all OCs. Of course again, this effect is more noticeable for lower pumping powers. The most important result is that the bifurcation border moves toward higher repetition rates with increasing the OC reflectivity. It should be mentioned that, for the pump power of 1090 W, the laser’s energy and pulse width are not significantly different for the OC reflectivities of 93 and 95%.

**Figure 3:**
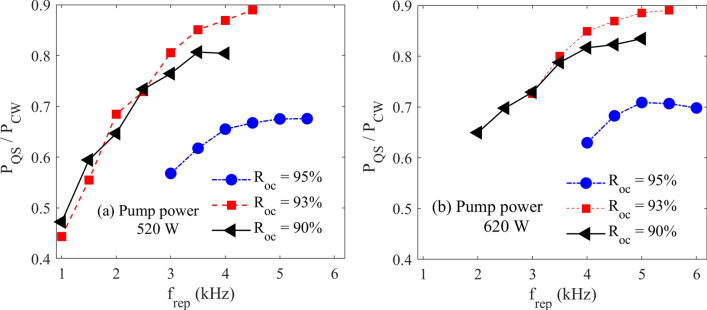
The pulsed to CW average power ratio versus repetition rate for different OCs and two pump powers of 520 and 620 W.

The pulsed to CW average power ratio is a key parameter indicating the capability of producing efficient pulses from the laser media. In Fig. 3, this parameter is compared for three OCs and two different pump powers. For all investigated OCs, the lost power decreases with the repetition rate, and the OC with a reflectance of 93% has a higher CW to pulse conversion ratio. We see that for both pump powers, the growth rate of this conversion ratio decreases at enough high repetition rates.

**Figure 4:**
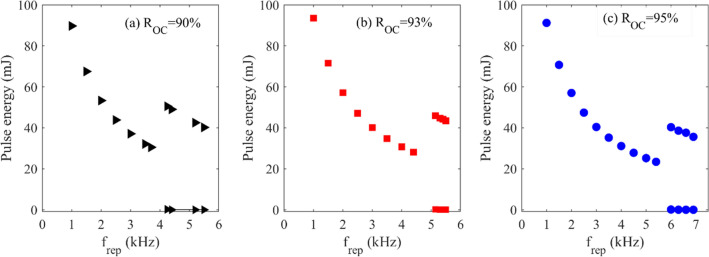
Numerical prediction of energy of the pulses in the pulse train versus the repetition rate for a pump power of 520 W and three different OC reflectivities.

The result of numerical simulation for a pump power of 520 W has been presented in Fig. 4. The used parameters were selected following the experimental conditions of Fig. 2a. The simulations reveal an irregular behavior in the pulse energy above specific frequencies. Above this specific repetition rate, the laser pulse train becomes unstable and multi-energy pulse output is observed. Similar to this behavior had been reported in actively Q-switched fiber lasers^29^, passively Q-switched solid-state lasers^37^ and regenerative amplifiers^38^.

Comparing the numerical predictions for the frequency range of stable operation of pulses with the experiments in Fig. 2 indicates that the theoretical and the experimental results are generally consistent. However, in the experiments, the bifurcation behavior is seen at slightly higher repetition rates than in the simulation. It should be mentioned that the laser works stably at higher repetition rates with OC reflectivity of 95% compared to the other OCs. According to the simulation results, at each pump power, the border of instability moves toward higher repetition rates if a higher OC reflectivity is used. However, the model predicts higher pulse energy values which is due to simplifications considered in the theoretical model such as neglecting the temperature dependency of the laser operation^39^, and the transverse distribution of the laser profile.

**Figure 5:**
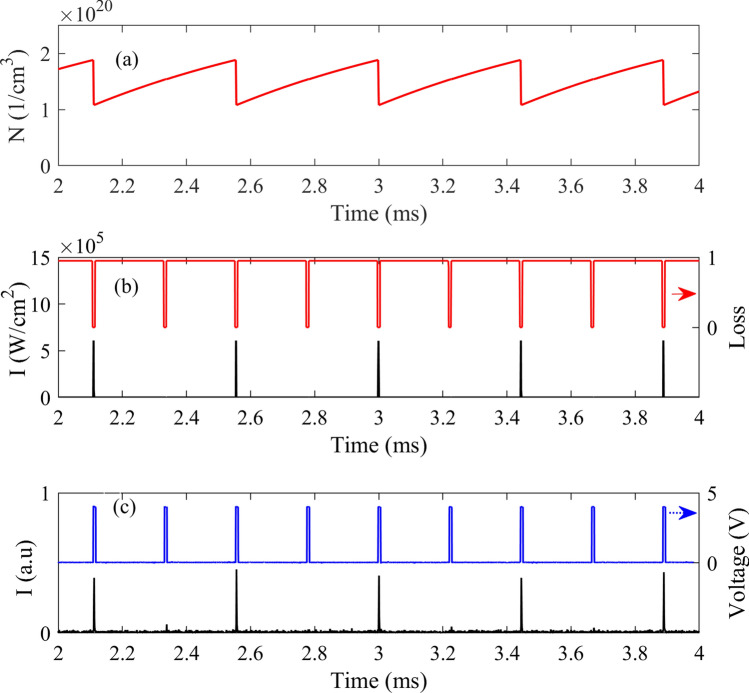
(**a**) The time variations of upper-laser-level population density, (**b**) the modulation loss and the calculated intra-cavity intensity, and (**c**) the measured laser output pulses in addition to a sample TTL voltage applied for driving the AO cell.

Figure 5 shows the simulation results for the laser output under the pump power of 520 W, a repetition rate of 4.5 kHz, and the R_OC_ of 90%. The population inversion density and the intra-cavity intensity, together with the measured photodiode signal for a similar laser experiment as in the simulation, are all shown in this figure. Also, the modulation loss of the resonator due to the Q-switching, in addition to the applied voltage on the AO modulator in the experiment, are depicted in the figure. Under these operational conditions, the laser produces two categories of pulses, in which the small pulses are emitted before the large pulses (the small pulses are not clearly seen in the scale of Fig. 5c). From Fig. 5c, a similar pulse train as what is seen in the simulation, is measured in the experiment, indicating good agreement between the theory and the experiment.

To achieve maximum average power from the laser under higher pump power densities, a compromise between OC reflectivity and maximum allowable repetition rate has to be found. The simulation results showed that at the pump power of 1100 W for OCs reflectivities of 90, 93 and 95%, the laser could be stably operated up to 10.40, 11.80 and 12.30 kHz, respectively. These values increase to near 11.90, 13.25 and 13.90 kHz, respectively, under the maximum pump power of 1220 W. Furthermore, under this pump power, the OC with 93% reflectivity produces maximum output power compared to other OCs. Based on these findings, many experiments were conducted under the pump powers up to 1220 W and for three different OCs, and the laser output characteristics were recorded.

**Figure 6:**
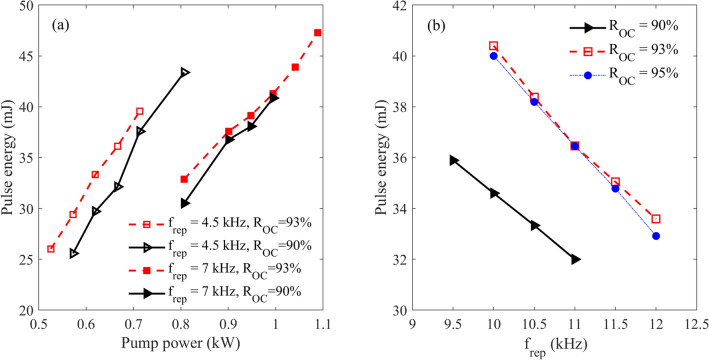
(**a**) The energy of output pulses as a function of a pump power density for different repetition rates and OC couplers. (**b**) The pulse energy as a function of repetition rate for different OCs and at the pump power of 1220 W.

Figure 6 shows the dependency of the measured pulse energy on the pump power (Fig. 6a) and the repetition rate (Fig. 6b) for three OCs reflectivities of 90, 93 and 95%. The results clearly show the dependency of the pulse stability border on the reflectivity of OCs. For example, as shown in Fig. 6b, in the case of 90% OC and the pump power of 1220 W, the bifurcation in pulse energy is observed near 11.0 kHz, while for 93% OC, this value is near 12.0 kHz.

According to the simulation and experimental results, the maximum average power of the laser was produced by OC with 93% reflectivity. Of course, using OC with a lower transmission under the stable operation of the laser has the drawback of efficiency decrement. In Fig. 6b, the dependency of pulse energy on f_rep_ was compared for different OCs under the maximum pump power of 1220 W, we see that both OCs of 93 and 95% have the same stability border determining the ultimate frequency of Q-switching. Since the laser energy for the 93% OC was maximum, the characteristics of the laser for this case were determined in details and the results are presented in the following.

**Figure 7:**
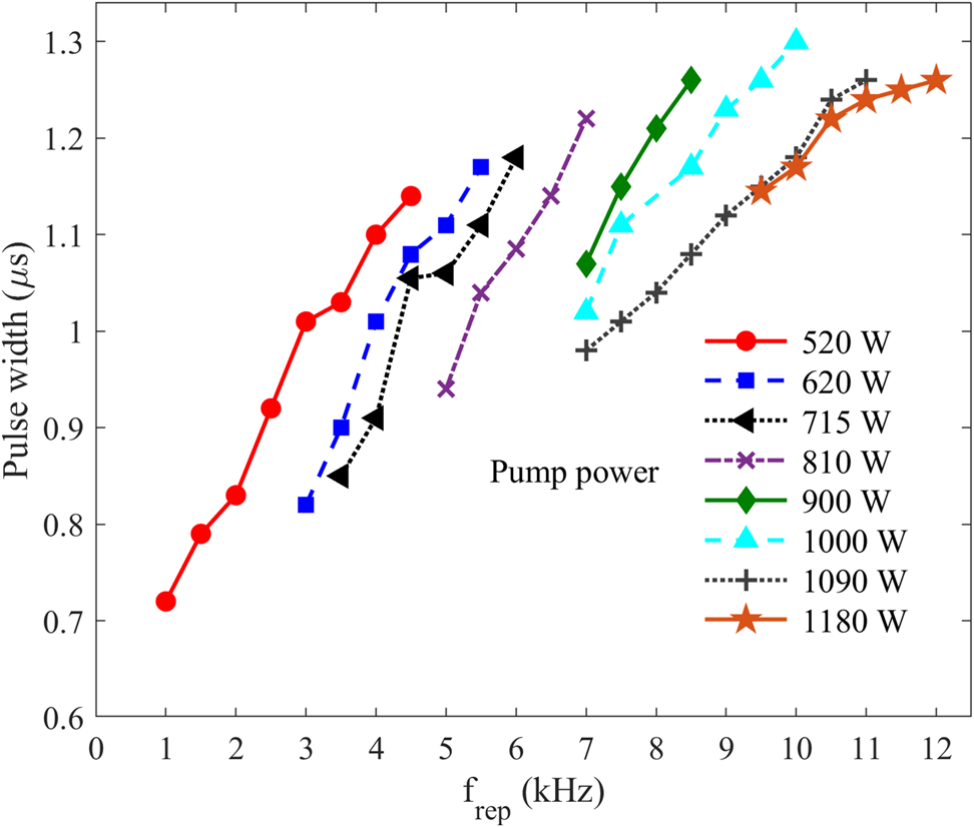
The variation of temporal width of the output pulses versus Q-switching repetition rate for various pump powers. The OC reflectivity was 93%.

Figure 7 shows the pulse width variations versus the Q-switching repetition rate for some pump powers for the OC reflectivity of 93%. As shown in this figure, the duration of the pulses is around one microsecond and increases with the repetition rate, particularly at lower repetition rates. The pulse width also decreases with the pump power. This behavior is noticeable at lower repetition rates, where the pulse energy is high. Indeed, when the incident pump power becomes less than saturation power, the pulse time duration strongly depends on the pump intensity. This is mainly due to the increase in the population inversion and decrease in the pulse rise time of Q-switching. For higher pump power levels, where the pumping reaches near the saturation, the pulse duration shows a smaller dependency on the repetition rate, which is a well-known behavior in Q-switched lasers. Actually, for the pump power greater than 1000 W, we see a moderate stop in increment of the pulse width for the repetition rates greater than 11.0 kHz.

Furthermore, it was observed that the pulse width changes with an increase in OC transmittance. However, the difference between values measured with the 93 and 95% OCs is insignificant. Notably, the observed trends are consistent with the previous reports on the behavior of pulse time duration under various deriving conditions of Q-switched lasers^13,17^. Noticeably, the maximum average power was achieved under the pump power of 1220 W and with a 93% output mirror.

**Figure 8:**
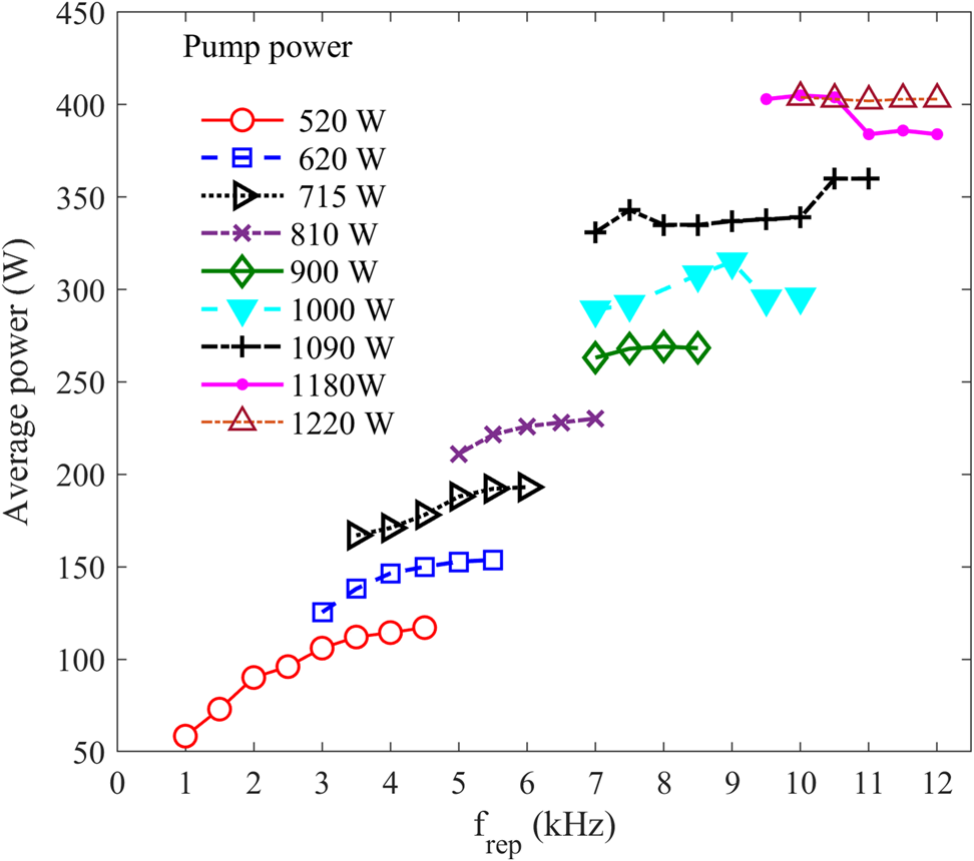
The average power of the laser versus the repetition rate for two different pump densities with R_OC_ of 93%.

Figure 8 shows the laser average power versus the repetition rate for various pump powers and with OC reflectivity of 93%. The maximum average power was obtained in two pump powers of 1180 and 1220 W. In the case of P_pump_ = 1180 W, between the repetition rates of 9.50 and 10.5 kHz, the average power was 403 W, and the laser pulses are stable for various repetition rates up to 10.5 kHz. However, above 10.5 kHz, the average power slightly decreased to around 380 W, and an unstable pulse regime was started. As the pump power was increased to 1220 W, the stable pulses were achieved up to the repetition rate of 12.0 kHz.

**Figure 9:**
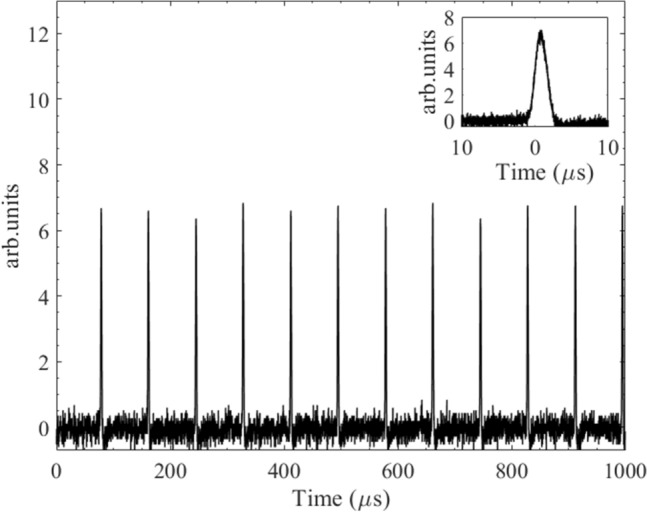
The laser output signal at f_rep_ of 12.0 kHz for the pump power of 1220 W and R_OC_ of 93%. Inset: single pulse time profile indicating the pulse duration is about 1.25 µs.

The maximum average power of 403 W seems to be a record obtained from a Q-switched thin-disk laser. The laser’s output stability is quite good in high repetition rates, in which the difference of the pulse amplitudes is less than ± 10%, as shown in Fig. 9.

## Conclusion

The design, optimization, and characteristics of an AO Q-switched Yb:YAG thin-disk laser with a new record of the maximum average power of 403 W were presented. The border of pulse energy instability was determined in both experiment and simulation for various operational conditions. In both cases, it was found that the laser could be stably operated at higher repetition rates if the value of output transmittance is controlled. Three OCs with the reflectance of 90, 93 and 95% were utilized to run the laser at higher frequencies and to maximize the average output power. The characteristics of laser output under various operational conditions have been determined.

The time duration of the output pulse was around one microsecond, which increases with repetition rate and decreases with pump power density. The maximum average power of 403 W has been recorded at a 12.0 kHz repetition rate and under pump power of 1220 W and OC with 93% reflectivity. The maximum pulse energy of 57 mJ was measured at the repetition rate of 1.00 kHz. Furthermore, the CW to pulsed power conversion increases with the repetition rate, with the highest value always occurring for the output reflectivity of 93%. The presented experimental results are consistent with the simulation results based on the rate equations.

The results of this study pave the way for developing a simple and economical high average power pulsed industrial laser based on TDL oscillators. Additionally, the results may be very promising in scaling up the average powers of Q-switched single oscillator solid-state lasers with good beam quality based on commercial thin-disk gain modules.

## Data Availability

All data generated or analysed during this study are included in this published article.
